# Pharmacological mechanisms of Ma Xing Shi Gan Decoction in treating influenza virus-induced pneumonia: intestinal microbiota and pulmonary glycolysis

**DOI:** 10.3389/fphar.2024.1404021

**Published:** 2024-08-05

**Authors:** Lin Jiang, Chen Bai, Jingru Zhu, Chen Su, Yang Wang, Hui Liu, Qianqian Li, Xueying Qin, Xiaohong Gu, Tiegang Liu

**Affiliations:** ^1^ College of Traditional Chinese Medicine, Beijing University of Chinese Medicine, Beijing, China; ^2^ Beijing Dingjitang Traditional Chinese Medicine Clinic Co., Ltd., Beijing, China; ^3^ Traditional Chinese Medicine Department, Beijing Jishuitan Hospital, Captial Medical University, Beijing, China; ^4^ Institute of Traditional Chinese Medicine for Epidemic Diseases, Beijing University of Chinese Medicine, Beijing, China; ^5^ Department of Respiratory Medicine, The First Clinical College of Beijing University of Chinese Medicine, Beijing, China

**Keywords:** H1N1 influenza virus, Ma Xing Shi Gan Decoction, intestinal microbiota, glycolysis, inflammatory response

## Abstract

**Background:**

Influenza virus is one of the most common pathogens that cause viral pneumonia. During pneumonia, host immune inflammation regulation involves microbiota in the intestine and glycolysis in the lung tissues. In the clinical guidelines for pneumonia treatment in China, Ma Xing Shi Gan Decoction (MXSG) is a commonly prescribed traditional Chinese medicine formulation with significant efficacy, however, it remains unclear whether its specific mechanism of action is related to the regulation of intestinal microbiota structure and lung tissue glycolysis.

**Objective:**

This study aimed to investigate the mechanism of action of MXSG in an animal model of influenza virus-induced pneumonia. Specifically, we aimed to elucidate how MXSG modulates intestinal microbiota structure and lung tissue glycolysis to exert its therapeutic effects on pneumonia.

**Methods:**

We established a mouse model of influenza virus-induced pneumoni, and treated with MXSG. We observed changes in inflammatory cytokine levels and conducted 16S rRNA gene sequencing to assess the intestinal microbiota structure and function. Additionally, targeted metabolomics was performed to analyze lung tissue glycolytic metabolites, and Western blot and enzyme-linked immunosorbent assays were performed to assess glycolysis-related enzymes, lipopolysaccharides (LPSs), HIF-1a, and macrophage surface markers. Correlation analysis was conducted between the LPS and omics results to elucidate the relationship between intestinal microbiota and lung tissue glycolysis in pneumonia animals under the intervention of Ma Xing Shi Gan Decoction.

**Results:**

MXSG reduced the abundance of Gram-negative bacteria in the intestines, such as Proteobacteria and *Helicobacter*, leading to reduced LPS content in the serum and lungs. This intervention also suppressed HIF-1a activity and lung tissue glycolysis metabolism, decreased the number of M1-type macrophages, and increased the number of M2-type macrophages, effectively alleviating lung damage caused by influenza virus-induced pneumonia.

**Conclusion:**

MXSG can alleviate glycolysis in lung tissue, suppress M1-type macrophage activation, promote M2-type macrophage activation, and mitigate inflammation in lung tissue. This therapeutic effect appears to be mediated by modulating gut microbiota and reducing endogenous LPS production in the intestines. This study demonstrates the therapeutic effects of MXSG on pneumonia and explores its potential mechanism, thus providing data support for the use of traditional Chinese medicine in the treatment of respiratory infectious diseases.

## 1 Introduction

In clinical practice, influenza virus pneumonia is a prevalent respiratory disease that affects approximately 5%–15% of the global population annually, causing 290,000 to 650,000 deaths (Moore et al., 2021). Type A H1N1 is one of the most common influenza viruses (Jiang et al., 2020). The influenza virus can trigger an excessive inflammatory response and an immune imbalance within the body, ultimately leading to a cytokine storm and severe damage to lung tissues, resulting in pneumonia. Currently, the prevention and treatment of pneumonia caused by influenza virus primarily rely on vaccines, antiviral medications, glucocorticoids, and symptomatic supportive care; however, owing to factors such as viral mutations and drug resistance development, the prevention and treatment of influenza virus pneumonia are limited.

Ma Xing Shi Gan Decoction (MXSG) is an ancient Chinese medicine prescription with a long history of use in treating pneumonia (Wang et al., 2011). It consists of four medicinal botanical drugs: *Ephedra sinica* Stapf (Ma Huang), seeds of *Prunus mandshurica* (Maxinm.) Koehne (Ku Xingren), the mineral medicine gypsum (Shi Gao), and *Glycyrrhiza glabra* L (Gan Cao). The names of all plants used in this article were verified at http://www.worldfloraonline.org on 16 October 2023. In clinical practice in China, MXSG has demonstrated significant efficacy, particularly in alleviating symptoms such as high fever, cough, wheezing, chest tightness, and chest pain associated with acute pneumonia. Recent studies on the pharmacological effects and mechanisms of MXSG have revealed that it can regulate the levels of inflammatory factors and pattern recognition receptors in the body (Ma et al., 2014; Shen and Yin, 2021); however, its specific mechanism of action remains unclear.

In recent years, treating respiratory diseases by modulating the gut microbiota has received increasing attention (Wang et al., 2022). Modulating and optimizing the composition of gut microbiota can serve as a potential therapeutic strategy to alleviate the severity and symptoms of viral pneumonia. Viral pneumonia can lead to an imbalance in gut microbiota and an increase in pathogenic bacteria (Gu et al., 2020). Preliminary research conducted by our team has identified irregularities in the gut microbiota structure during pneumonia (Bai et al., 2020). The gut microbiota plays a significant role in regulating host glycolysis. Research has demonstrated that intestinal *Akkermansia muciniphila* and its metabolite pentadecanoic acid can inhibit glycolysis by antagonizing the activity of FUBP1 (Xu et al., 2024). Glycolysis, one of the primary energy metabolism pathways in organisms, plays a critical role in regulating the inflammatory response. For instance, immune cells primarily rely on glycolysis to provide the energy required during the peak of inflammation (Soto-Heredero et al., 2020); Influenza virus can exacerbate inflammatory responses in lung tissue by promoting glycolysis in macrophages (Zhu et al., 2021). HIF-1α regulates the immune metabolic phenotype of macrophages (Evans et al., 2021). Herbal treatment in influenza A has the effect of regulating gut microbiota, reducing viral load in the lungs, and alleviating inflammatory damage (Deng et al., 2021; Liu et al., 2022). *Ephedra sinica* Stapf polysaccharide extracted from *E. sinica* Stapf can enhance the abundance of *Lactobacillales* and Bifidobacteriaceae, potentially indicating a significant target for the treatment of influenza virus infection (Liu et al., 2020). However, whether MXSG exerts its therapeutic effects on pneumonia by regulating the “gut microbiota-lung glycolysis” axis requires further investigation.

Therefore, we aimed to establish a mouse model of influenza virus-induced pneumonia through intranasal infection with type A Hemagglutinin 1 Neuraminidase 1 (H1N1) influenza virus. MXSG was administered orally. By observing the therapeutic effects of MXSG on lung damage in the animal model and its impact on gut microbiota structure and lung glycolysis, we aimed to elucidate the mechanism underlying the treatment effect of MXSG against influenza virus-induced pneumonia.

## 2 Materials and methods

### 2.1 Animals and viruses

*Experimental animals*: We purchased 50 male BALB/c mice aged 6–8 weeks and weighing 20 ± 2 g from Sibeifu (Beijing) Biotechnology Co., Ltd. [License No. SCXK (Jing) 2019–0010], under animal batch number 110324221105336075. They were raised in the P2 laboratory of the Experimental Animal Center of the Chinese Center for Disease Control and Prevention [License No. SYXK (Jing) 2020–0033]. All animal experiments were conducted in accordance with the guidelines of Animal Welfare and Ethics of the Institutional Animal Care and Use Committee. The protocols described in the present study received approval from the Animal Care and Use Committee of the Chinese Center for Disease Control and Prevention of Viral diseases (approval ID: 20220624076).

*Virus strain*: The mouse lung-adapted strain of type A H1N1 influenza virus (A/PR/8/34) was provided by the Chinese Center for Disease Control and Prevention, with an LD50 of 1 × 10^−4.5^/50 µL. The mice were intranasally infected with influenza virus (A/PR/8/34) at 50 μL of l×LD50/mouse or 50 μL of saline.

### 2.2 Drugs

MXSG was prepared in accordance with the standard of “Pharmacopoeia of the People’s Republic of China.” Each prescription consisted of *E. sinica* Stapf (Lot no.: 2110012S, 9 g), seeds of *P. mandshurica* (Maxinm.) Koehne (Lot no.: 2110006S, 9 g), the mineral medicine gypsum (Lot no.: 2110001S, 18 g), and *G. glabra* L (Lot no.: 2109018C, 6 g). The formula was prepared as granules (Huaren Sannine, Ltd), with a granules to crude botanical drugs ratio of 1:8.75. Oseltamivir phosphate capsules [Batch No. M1050] at 75 mg oseltamivir × 10 capsules were produced by Roche Pharmaceuticals, Ltd., Italy. 2-Deoxy-d-glucose (2DG) [Batch No. 1758, 9500] at 1 g was produced by INALCO.

The dosages of the drugs were converted according to the standard body weight of adults and the mouse dose conversion factor of 9.1. MXSG granules and oseltamivir phosphate capsules were fully dissolved in distilled water before use, and suspensions with concentrations of 72.8 mg/mL and 2.5 mg/mL were administrated once a day by oral gavage, respectively. The glycolysis inhibitor 2DG was fully dissolved in physiological saline to prepare a solution with a concentration of 50 mg/mL for intraperitoneal injection.

### 2.3 Grouping, modeling, and administration

After 1 week of adaptive feeding, the mice were randomly divided into five groups, each consisting of 10 mice: control group, influenza virus infection group (model group), MXSG treatment group (MXSG group), oseltamivir phosphate treatment group (oseltamivir group), and 2DG treatment group (2DG group). Owing to its competitive inhibition of hexokinase, 2DG, a glucose analog, is often employed as a glycolysis inhibitor (Barban and Schulze, 1961; Pelicano et al., 2006). Except for the control group, the other mice were intranasally inoculated with 50 µL of virus fluid containing 1 unit of LD50 under mild isoflurane anesthesia to establish the model. The control group received the same method but with 50 µL of phosphate-buffered saline administered intranasally.

Starting from the second day after modeling, the Chinese medicine treatment group received MXSG at 0.2 mL (i.g.), the oseltamivir group received oseltamivir phosphate capsules at 0.2 mL (i.g.), and the 2DG group received 2DG at 0.2 mL (i.p.) once daily for six consecutive days. Both the control group and model group received an equivalent volume of distilled water by oral gavage.

### 2.4 Ma Xing Shi Gan Decoction identification detected by UPLC-MS/MS

The concentrated water of Ma Xing Shi Gan Decoction are freeze-dried by vacuum freeze-dryer (Scientz-100F). Dissolve 100 mg lyophilized powder in 1.2 mL 70% methanol. Vortex for 30 s, repeating five more times every 30 min. Chill the sample overnight at 4°C. The UPLC-ESI-MS/MS system was utilized to analyze the sample extracts (UPLC: SHIMADZU Nexera X2 www.shimadzu.com.cn/; MS, Applied Biosystems 4500 Q TRAP, www.appliedbiosystems.com.cn/). The analytical parameters were optimized as follows: Agilent SB-C18 column (1.8 µm, 2.1 × 100 mm) was employed, with a mobile phase comprising solvent A (pure water +0.1% formic acid) and solvent B (acetonitrile +0.1% formic acid). A gradient elution program was initiated at 95% A and 5% B, linearly transitioning to 5% A and 95% B over 9 min, held for 1 min, then adjusted back to 95% A and 5% B within 1.10 min, maintained for 2.9 min. The flow rate was 0.35 mL/min, column temperature set at 40°C, and injection volume was 4 μL. The effluent was directed to an ESI-QTRAP-MS for analysis. LIT and QQQ scans were performed on the AB4500 Q TRAP UPLC/MS/MS System, a triple quadrupole-linear ion trap mass spectrometer equipped with an ESI Turbo Ion-Spray interface. Operating in both positive and negative ion modes, the system was controlled by Analyst 1.6.3 software from AB Sciex. Key ESI source settings included a turbo spray ion source at 550°C, ion spray voltages of 5500 V (positive) and −4500 V (negative), and optimized gas flows for source gas I (50 psi), gas II (60 psi), and curtain gas (25.0 psi). High collision-activated dissociation (CAD) was employed. Instrument tuning and calibration were conducted using polypropylene glycol solutions at 10 and 100 μmol/L, respectively, in QQQ and LIT modes.

### 2.5 Sample collection and detection

On the seventh day after virus infection, which corresponded to the sixth day of treatment, the mice were weighed and then collected for heart blood samples under isoflurane anesthesia. EP tubes were used to collect blood samples, which were then centrifuged, and the upper serum samples were collected and stored in a freezer at −80°C. After weighing the mouse lungs, the left lung was immersed in a 4% paraformaldehyde solution, and the right lung and cecal content samples were placed in cryotubes and stored in a −80 °C freezer.

#### 2.5.1 Lung index and histopathological analysis

The method of calculating the lung index is as follows: lung mass/body mass × 100%. The lung tissue injury was observed by histopathology. The fixed lung tissues were dehydrated and embedded, cut into 0.5 µm sections, and subjected to hematoxylin-eosin (HE) staining. Lung tissue pathological morphology was observed under a 200× light microscope. A pathologist blinded to the treatment conducted the histological assessments. The score was graded according to the sum of the score for degree of damage such as hemorrhage, the number of infiltration cells, and edema. Each histological characteristic was assigned a score ranging from 0 to 3.

#### 2.5.2 ELISA detection of inflammatory factors and LPS levels

Each group was randomly selected, and six samples were chosen. Lung samples were homogenized using a homogenizer and centrifuged at 5000 rpm for 10 min. Serum was obtained by centrifuging blood samples at 4°C and 3000 rpm for 15 min. Tumor necrosis factor (TNF)-α, interleukin (IL)-1β, IL-6, IL-10, and lipopolysaccharide (LPS) levels were detected using an MS Mouse TNF-α ELISA Kit (Catalog No. E-MSEL-M0002, Elabscience), MS Mouse IL-1β ELISA Kit (Catalog No. E-MSEL-M0003, Elabscience), MS Mouse IL-6 ELISA Kit (Catalog No. E-MSEL-M0001, Elabscience), MS Mouse IL-10 ELISA Kit (Catalog No. E-MSEL-M0031, Elabscience), and Mouse (LPS) ELISA Kit (Catalog No. CSB-E13066m, Huamei).

#### 2.5.3 Targeted metabolomics analysis of lung tissue glycolysis levels

Lung tissue samples from six randomly selected mice from each group were used. Approximately 80 mg of lung tissue was combined with 200 µL of distilled water and homogenized using a FastPrep-24 5G homogenizer. Afterward, 800 µL of methanol-acetonitrile solution (1:1, v/v) was added, followed by 10 µL succinic acid-d6 (internal standard). The mixture was vortexed for 60 s, sonicated twice at low temperature for 30 min each, then placed in a −20 °C freezer for 1 h to precipitate proteins. After centrifugation at 4°C and 14000 × g for 20 min, the supernatant was collected and freeze-dried. The samples were separated using an Agilent 1290 Infinity LC system and analyzed using a 5500QTRAP mass spectrometer (AB SCIEX). Metabolites were identified using Multiquant software, which extracted chromatographic peak areas and retention times. Retention times were corrected using standard substances of energy metabolites (Robinson et al., 2020). The differential metabolites in glycolysis were screened by targeting metabolomics in lung tissue.

#### 2.5.4 Lactate production

Measurement of lactate concentration was utilized with a lactate Assay kit (A019-2-1, Nanjing Jiancheng Bioengineering Research Institute) according to the manufacturer’s protocol. The main reaction mixture contains a 2 μL sample solution, 20 μL enzyme working solution, and 20 μL color developer. The sample was incubated at 37°C for 10 min, and the absorbance was measured at wavelength of 530 µm.

#### 2.5.5 Western blot for lung tissue proteins

For each group, lung tissue samples from three randomly selected mice were used. Lung tissue samples were thawed, lysed, and homogenized with RIPA lysis buffer to extract proteins. Protein concentration was determined using the bicinchoninic acid method. Uniform amounts of protein were subjected to SDS-polyacrylamide gel electrophoresis, transferred onto PVDF membranes, and blocked with 5% skim milk at room temperature for 2 h. The membranes were then probed with primary antibodies against GAPDH (1:1000), NP (1:2000), MPO (1:3000), GLUT1 (1:1000), HK2 (1:10,000), PKM2 (1:1000), LDHA (1:5000), HIF-1α (1:1000), CD80 (1:2000), and CD206 (1:2000) overnight at 4°C. After three consecutive 10-min washes with TBST, the membranes were incubated with secondary antibodies at room temperature for 90 min. Following three additional TBST washes for 10 min each, the membranes were subjected to an ECL reaction, and images were captured. ImageJ software was used to analyze the grayscale values and quantify the protein expression levels of myeloperoxidase (MPO), glucose transporters1 (GLUT1), hexokinase II (HK2), pyruvate kinase 2 (PKM2), lactate dehydrogenase A (LDHA), hypoxia inducible factor-1 (HIF-1α), cluster of differentiation 80 (CD80), and cluster of differentiation 206 (CD206) in lung tissue.

#### 2.5.6 16S rRNA gene sequencing of gut microbiota structure

For each group, cecal contents from six randomly selected mice were used. The cetyltrimethylammonium bromide (CATB) method was employed to extract genomic DNA from the samples, and the purity and concentration of DNA were assessed through 1% agarose gel electrophoresis. The V3+V4 variable region was PCR-amplified using primers 341F (5′-CCTAYGGGRBGCASCAG-3′) and 806R (5′-GGACTACNNGGGTATCTAAT-3′). Library construction was carried out using the NEB Next Ultra DNA Library Prep Kit, and the constructed libraries were quantified using an Agilent 5400 and Q-PCR. After library qualification, sequencing was performed on a NovaSeq 6000 sequencer. Quality control, denoising, merging, and operational taxonomic unit (OTU) generation were performed using the DADA2 plugin in QIIME2. Representative sequences of ASVs were aligned to the Greengenes Database using the QIIME2 feature-classifier plugin to obtain species annotation information (DeSantis et al., 2006). The proportions of sequence numbers at each group’s phylum and genus levels were calculated based on OTU absolute abundance and annotation information. Alpha and beta diversity analyses were mainly completed using the QIIME2 diversity plugin. Chao and Shannon indices were analyzed using the Wilcox Test method, and Principal Coordinates Analysis (PCoA) was performed using the Weighted Unifrac method. Linear Discriminant Analysis Effect Size (LDA) was employed to analyze the variation in microbial groups between the groups, with a discriminant feature threshold of 2.0.

#### 2.5.7 Immunofluorescence detection of macrophage markers in lung tissue

Paraffin embedding was performed on three random lung tissue samples from each group. Antigen retrieval of lung tissue sections was performed using EDTA buffer (0.01M, pH 9.0), followed by blocking with normal goat serum for 30 min. The sections were incubated overnight at 4 °C with primary antibodies against F4/80 (1:200), CD206 (1:100), and CD80 (1:100). After washing with PBST, the sections were incubated with fluorescent (Cy3)-labeled goat anti-mouse IgG (1:100) and fluorescent (FITC)-labeled goat anti-rabbit IgG for 1 h the following day. Finally, DAPT was added and left to incubate in the absence of light for 5 min. Finally, a fluorescence microscope was used to observe and document the fluorescence.

#### 2.5.8 Detection of M1/M2 macrophage markers by flow cytometry

For each sample, 100 μL of cell suspension was taken and mixed with 1 μL of each fluorescently labeled antibody, including CD45FITC, CD11bV450, F4/80 PE-Cy7, CD86PE, and CD206PE. The mixture was incubated at room temperature in the dark for 20 min. Subsequently, the cells were mixed with PBS and centrifuged at 300 *g* for 5 min, with this step being repeated twice. Finally, the cells were resuspended in 2 mL of PBS in a tube and analyzed using the Cytek NL-CLC3000 flow cytometer.

### 2.6 Data analysis

Experimental data are expressed as mean ± SD. Statistical analysis was performed using Prism 8 (GraphPad, CA, United States), with one-way ANOVA used for comparisons between multiple groups. Spearman’s correlation analysis was used for correlation testing. Statistical significance is reported at *P* < 0.05.

## 3 Results

### 3.1 Characterization of chemical metabolites in Ma Xing Shi Gan Decoction

**FIGURE 1:**
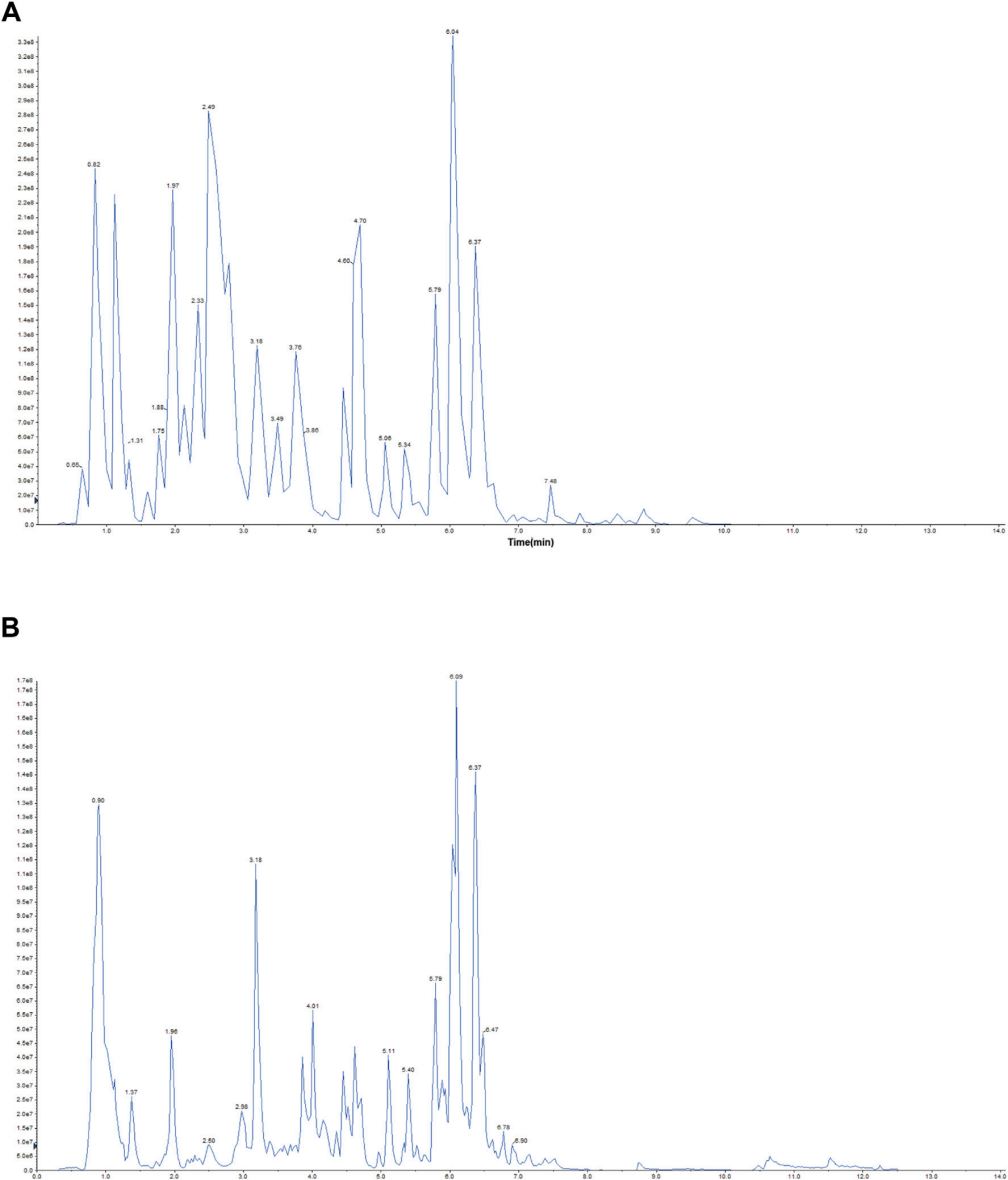
Representative total ion chromatogram MXSG. **(A)** Positive ion chromatogram of MXSG UPLC-MS/MS. **(B)** Anion chromatogram of MXSG UPLC-MS/MS.

Representative total chromatographic peaks obtained by UPLC-MS/MS (Figure 1). The primary and secondary spectrum data of mass spectrometry were qualitatively analyzed using the MetWare database and the published metabolite information database. Consequently, 96 elements were identified from MXSG. Table 1 presents the relative content over 10,000,000, and the top 18 chemical metabolites included 9 flavonoids, 6 alkaloids, 2 phenolic acids, and 1 terpenoids. The highest relative content was ephedrine and reached 740,220,000.

**TABLE 1:** Top 18 chemical metabolites in MXSG detected by UPLC-MS/MS.

Index	Metabolites	Formula	Adduct	Relative content	Category
pmp000723	Ephedrine	C10H15NO	+H	740220000	Alkaloids
pmp000725	Methylephedrine	C11H17NO	+H	658490000	Alkaloids
mws1580	Amygdalin	C20H27NO11	-H	217213333	Alkaloids
pmp000724	Pseudoephedrine	C10H15NO	+H	161333333	Alkaloids
pmp000722	Norephedrine	C9H13NO	+H	139016667	Alkaloids
mws1073	Apigenin6,8-C-diglucoside	C27H30O15	+H	91153333	Flavonoids
pme3227	Vitexin 2″-O-β-L-rhamnoside	C27H30O14	+H	89447333	Flavonoids
pmp000405	Isoglycyrrhizic acid	C42H62O16	+H	79180667	Terpenoids
pmp001287	N-Benzylmethylene isomethylamine	C8H9N	+H	67310333	Alkaloids
pmp000417	Daidzein-4′-glucoside	C21H20O9	+H	30344667	Flavonoids
pme1587	Daidzein-7-O-glucoside	C21H20O9	+H	29643333	Flavonoids
pmp000550	Calycosin-7-glucoside	C22H22O10	+H	27922000	Flavonoids
mws2212	Caffeic acid	C9H8O4	-H	19148333	Phenolic acids
mws0902	Liquiritigenin	C15H12O4	-H	18927667	Flavonoids
pmn001419	1-O-[(E)-p-Cumaroyl]-β-D-glucopyranose	C15H18O8	-H	16579000	Phenolic acids
pmp000116	Apigenin-8-C-glucoside	C21H20O10	+H	13044333	Flavonoids
mws0024	Gallic acid	C7H6O5	-H	12794667	Flavonoids
GQ512006	Quercetin-3-O-glucoside-7-O-rhamnoside	C27H30O16	+H	12016667	Flavonoids

### 3.2 Improvement in inflammatory state of influenza virus-induced pneumonia mice by MXSG

**FIGURE 2:**
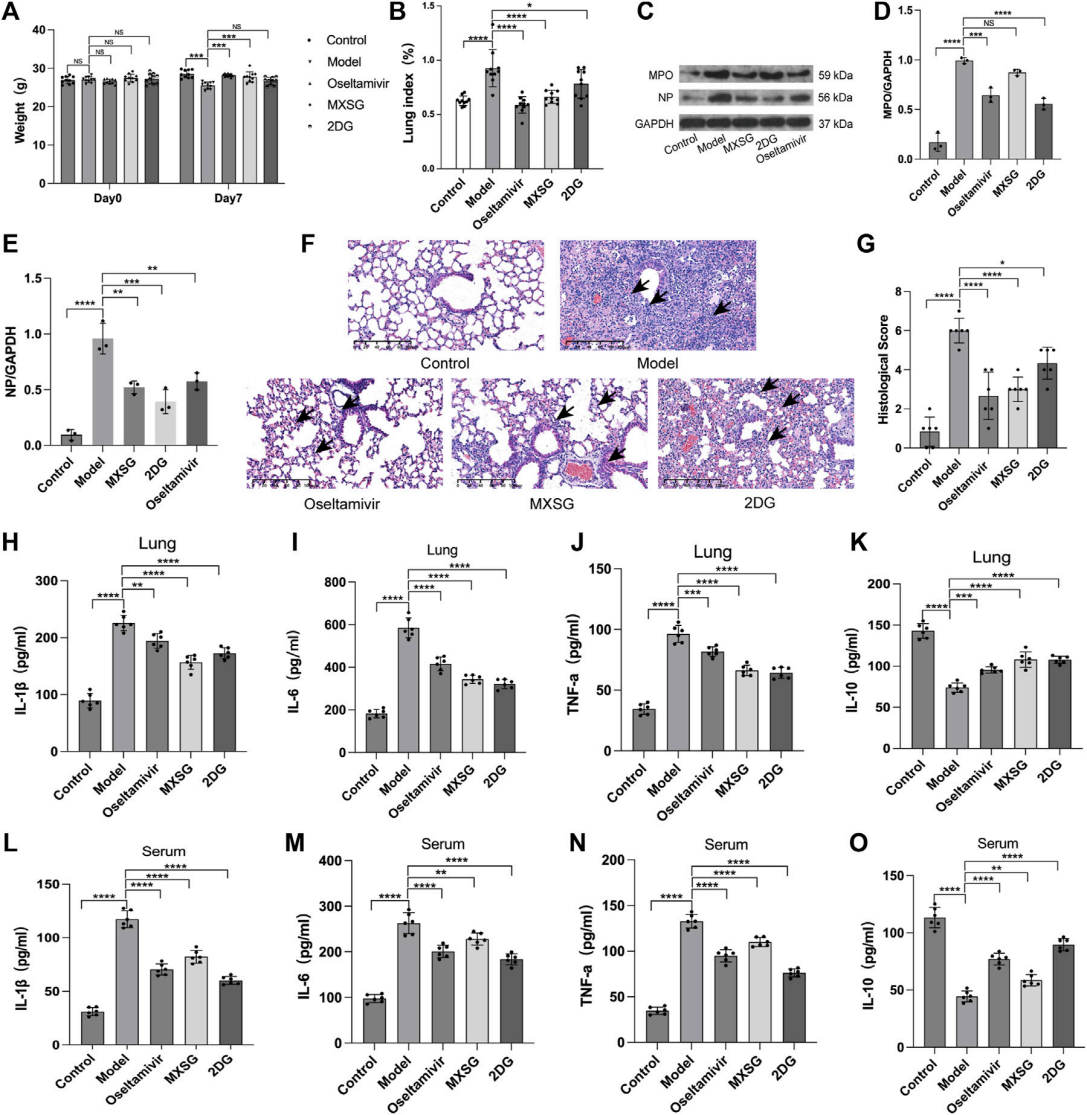
Inflammatory state of the model animals. **(A)** Animal body weight (n = 10). **(B)** Animal lung weight index (n = 10). **(C–E)** Protein imprinting analysis of MPO and NP in lung tissue (n = 3). **(F)** Animal lung tissue pathology (hematoxylin and eosin staining, 20×). Inflammatory cell infiltration in alveoli (black arrow). **(G)** Quantified scoring of pulmonary pathology (n = 6). **(H)** Lung IL-1β (n = 6). **(I)**, Lung IL-6 (n = 6). **(J)** Lung TNF-α (n = 6). **(K)** Lung IL-10 (n = 6). **(L)** Serum IL-1β (n = 6). **(M)** Serum IL-6 (n = 6). **(N)** Serum TNF-α (n = 6). **(O)** Serum IL-10 (n = 6). *P < 0.05, **P < 0.01, ***P < 0.001, ****P < 0.0001.

Compared to the control group, on the seventh day of modeling, the model group exhibited a decrease in body weight (Figure 2A) and an increase in the lung weight index (Figure 2B), and HE staining revealed damaged lung cell membranes, loss of alveolar tissue structure, thickened alveolar walls, and substantial infiltration of inflammatory cells in the alveolar cavity (Figure 2F). The lung pathological scores were higher (Figure 2G). The protein expression of MPO, NP increased (Figures 2C–E). The levels of inflammatory factors IL-1β, IL-6, and TNF-α were increased (Figures 2H–J, L–N), whereas IL-10 was reduced (Figure 2K, O). These findings indicated the successful establishment of the influenza virus-induced pneumonia model.

Compared to the model group, both the MXSG and oseltamivir groups showed an increase in body weight (Figure 2A) and a decrease in lung weight index (Figure 2B) on the seventh day of modeling. The lung morphology of the animals in these groups was intact, with fewer infiltrating inflammatory cells in the alveoli and significant improvement in lung tissue pathology (Figure 2F). The lung pathological scores were lower (Figure 2G). The expression of MPO, NP protein decreased (Figures 2C–E). Serum inflammatory factors IL-1β, IL-6, and TNF-α decreased (Figure 2H–J, L–N), whereas IL-10 increased (Figure 2K, O). This suggests that MXSG can effectively treat influenza virus-infected pneumonia, inhibit viral replication, and alleviate systemic inflammatory responses. Its efficacy is comparable to oseltamivir.

The 2DG group exhibited a lower lung weight index (Figure 2B) than that of the model group. The lung alveolar morphology was relatively intact, with thicker alveolar walls and increased infiltration of inflammatory cells in the alveolar cavity (Figure 2F). The lung pathological scores were lower (Figure 2G). The expression of MPO, NP protein decreased (Figures 2C–E). Inflammatory factors IL-1β, IL-6, and TNF-α decreased (Figure 2H–J, L–N), whereas IL-10 increased (Figure 2K, O). This suggests that inhibiting lung tissue glycolysis can suppress viral replication and improve the inflammatory response in influenza virus-induced pneumonia mice.

### 3.3 Effects of MXSG on glucose metabolism in influenza virus-induced pneumonia mice

**FIGURE 3:**
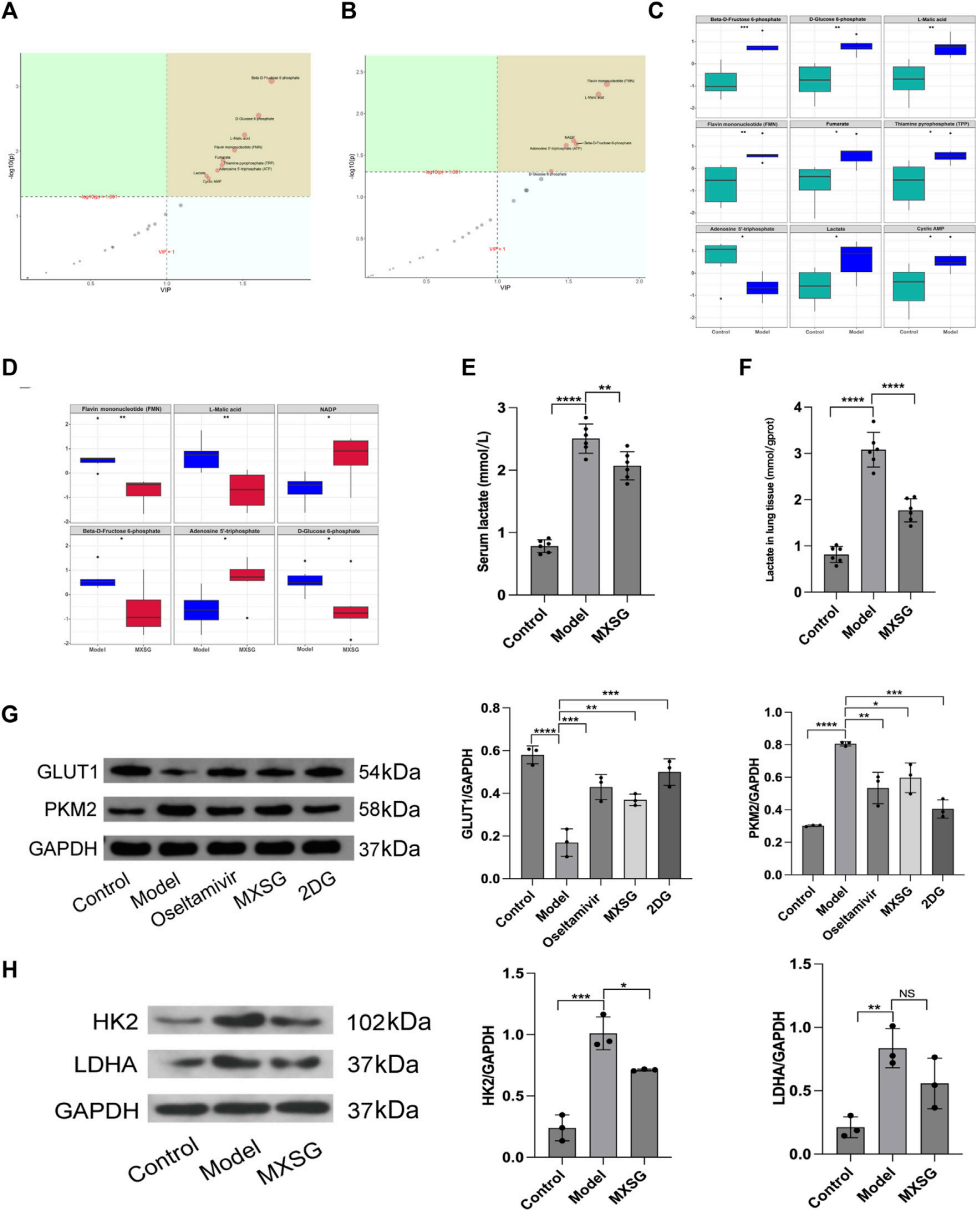
Lung tissue glucose metabolism levels and key glycolytic protein expression. **(A)** PLS-DA importance plot of metabolites between the control and model groups (n = 6). **(B)** PLS-DA importance plot of metabolites between the model and MXSG groups (n = 6). **(C)** Box plot of differential metabolites between the control and model groups (n = 6). **(D)** Box plot of differential metabolites between the model and MXSG groups (n = 6), **(E)** Lactate concentration in serum (n = 6). **(F)** Lactate concentration in lung tissues (n = 6). **(G)** Protein imprinting analysis of PKM2 and GLUT1 in lung tissues (n = 3). **(H)** Protein imprinting analysis of LDHA and HK2 in lung tissues (n = 3). *p < 0.05, **p < 0.01, ***p < 0.001, ****p < 0.0001.

The treatment of influenza virus-infected pneumonia with MXSG is effective, and glycolysis inhibitors can alleviate inflammation by regulating lung tissue glycolysis. Therefore, we further examined the metabolic levels and key regulatory proteins of lung tissue glycolysis after treatment with MXSG. Targeted metabolomics results indicated that, compared to the control group, the model group had increased levels of glucose-6-phosphate, fructose-6-phosphate, lactate, malic acid, flavin mononucleotide (FMN), fumarate, thiamine pyrophosphate (TPP), cyclic adenosine monophosphate (cAMP), and the levels of adenosine triphosphate (ATP) decreased (Figure 3A, C). Compared to the model group, the MXSG group showed decreased levels of glucose-6-phosphate, fructose-6-phosphate, FMN, and malic acid. In contrast, the levels of nicotinamide adenine dinucleotide phosphate (NADP+) and ATP increased (Figure 3B, D). Consistently, Using biochemical methods to detect lactate levels, the results showed that serum lactate and lactate in lung tissue homogenates were significantly higher in the model group compared to the control group (Figure 3E, F); MXSG can reduce the level of lactate (Figure 3E, F). Additionally, we examined glycolysis-related proteins. Compared to the control group, the model group exhibited decreased levels of GLUT1 protein but increased levels of HK2, PKM2, and LDHA proteins (Figure 3G, H); however, MXSG reversed this phenomenon (Figure 3G, H). An equivalent amount of glucose produced less ATP through glycolysis compared to oxidative phosphorylation. Our results showed an increase in glycolysis-related products in the lung tissues of influenza virus-induced pneumonia mice, a decrease in ATP, and an increase in glycolytic enzyme expression. MXSG reversed these effects, suggesting that lung tissue glucose metabolism in influenza virus-induced pneumonia mice may shift from oxidative phosphorylation to glycolysis, and MXSG may alter the glucose metabolism pattern in pneumonia by transitioning lung tissue glucose metabolism from glycolysis to oxidative phosphorylation.

### 3.4 Effect of MXSG on the gut microbiota composition in influenza virus-induced pneumonia mice

**FIGURE 4:**
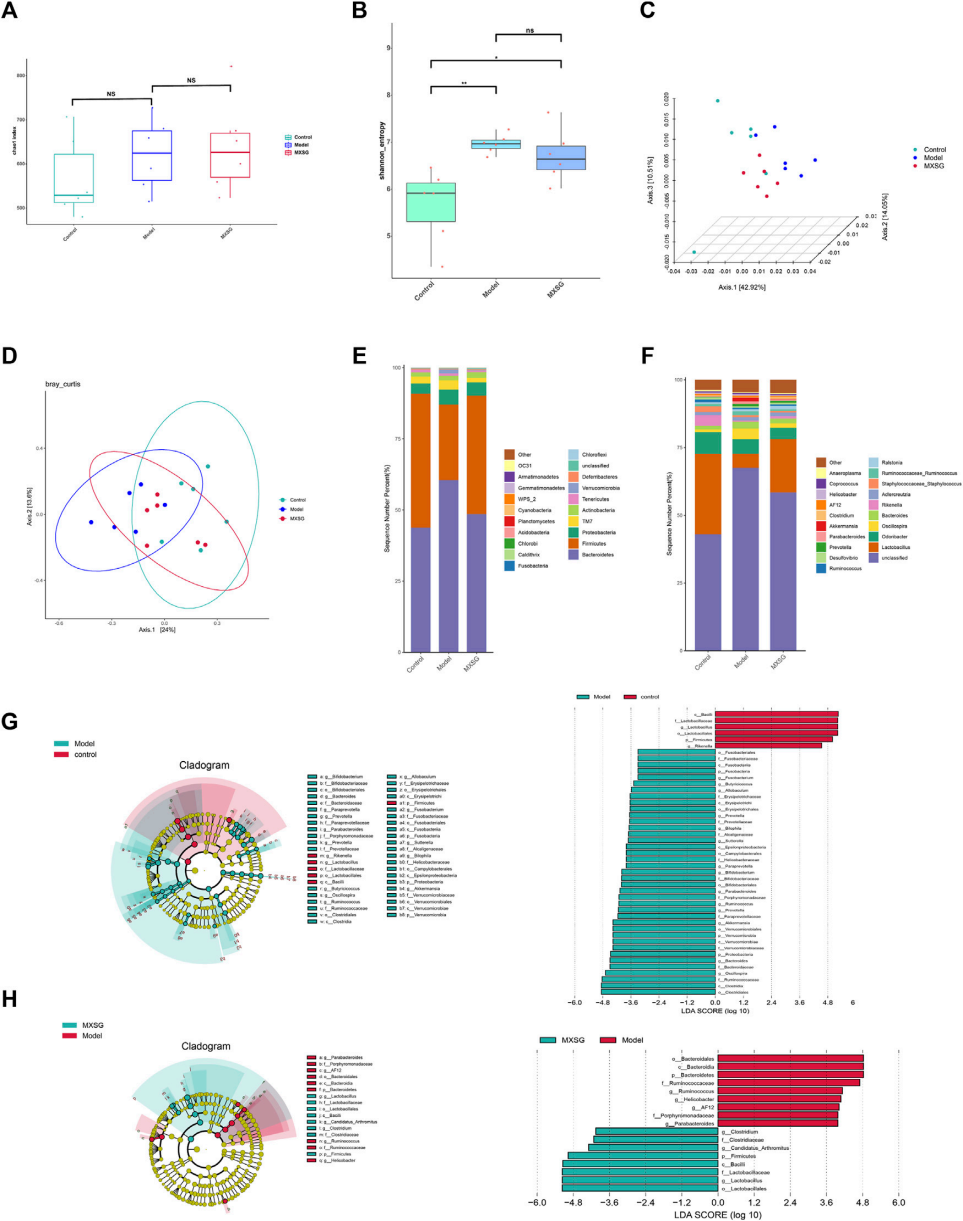
Changes in gut microbiota (n = 6). **(A)** Chao1 index. **(B)** Shannon index. **(C, D)** PCoA based on Weighted Unifrac distance. **(E)** Community distribution of gut microbiota at the phylum level. **(F)** Community distribution of gut microbiota at the genus level. **(G)** LEfSe analysis of different biomarkers and the histogram of the distribution of LDA values between the control and model groups. **(H)** LEfSe analysis of different biomarkers and the histogram of the distribution of LDA values between model and MXSG groups. *P < 0.05, **P < 0.01.

Viral pneumonia can lead to imbalances in the gut microbiota, and the diversity and richness of gut microbiota can affect the body’s immune function. Regulating the immune response can alleviate viral pneumonia by improving the gut microbiota (Cox and Tregoning 2018; Jung et al., 2017). Therefore, the effect of MXSG on the gut microbiota was observed by 16S rRNA gene sequencing. The results indicated that there was no notable disparity in the Chao1 index among the control, model, and MXSG groups (Figure 4A); however, the Shannon index of the model group surpassed that of the control group (Figure 4B). The PCoA showed that the control and model groups were dispersed, with a significant shift in the MXSG group (Figures 4C, D). This indicates that the gut microbiota of influenza virus-induced pneumonia mice undergoes changes, and MXSG can adjust its gut microbiota composition.

To further explore the differences in gut microbiota among the control, model, and MXSG groups, we analyzed the differential species of gut microbiota at the phylum and genus levels. At the phylum level, the model group displayed characteristic changes compared to the control group, with a rise in the prevalence of Bacteroidetes and Proteobacteria, along with a decline in the abundance of Firmicutes. MXSG reversed this trend (Figure 4E). At the genus level, the model group showed reduced levels of *Lactobacillus* and *Rikenella* and increased levels of *Fusobacterium, Butyricicoccus, Allobaculum, Paraprevotella, Parabacteroides, Ruminococcus, Prevotella, Akkermansia, Oscillospira, Bacteroides, Bilophila, Sutterella, and Bifidobacterium* compared to the control group (Figures 4F, G). Based on the significant changes in the gut microbiota of influenza virus-induced pneumonia mice, MXSG increased the levels of *Lactobacillus*, *Candidatus Arthromitus*, and *Clostridium* and decreased the levels of *AF12*, *Helicobacter*, *Ruminococcus*, and *Parabacteroides* (Figures 4F, H). These results suggest that MXSG can reduce the abundance of pathogenic bacteria and increase the abundance of beneficial bacteria.

### 3.5 Association analysis between gut microbiota and glycolysis

Based on the results of gut microbiota detected by 16S rRNA gene sequencing, viral infection can increase the abundance of Gram-negative bacteria, such as *Parabacteroides*, Prevotellaceae*_Prevotella*, and *Bacteroides* in the Bacteroidetes and Proteobacteria phyla, while MXSG can reduce the abundance of Gram-negative bacteria, such as *Helicobacter* and *Parabacteroides*. Gram-negative bacteria’s cell walls are mainly composed of LPS and have a certain pathogenicity, which can induce immune cell glycolysis reprogramming and thus regulate the body’s inflammatory response (Mills et al., 2016; Pan et al., 2022).

**FIGURE 5:**
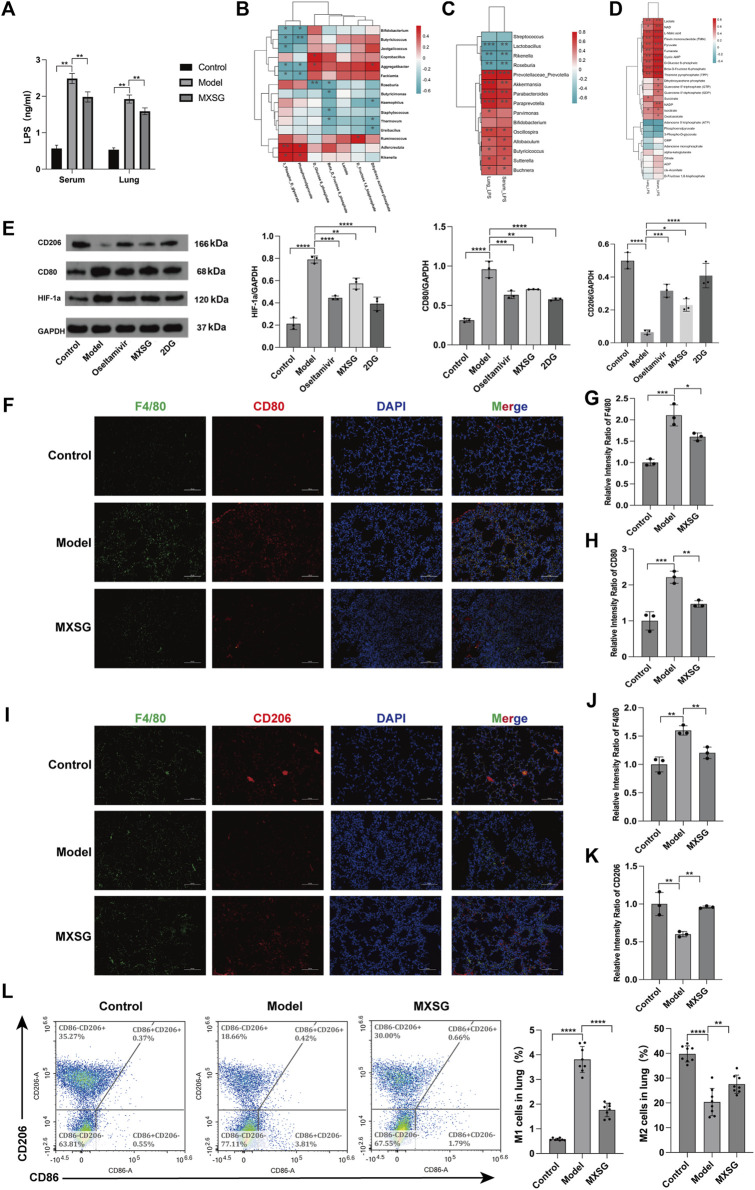
Correlation analysis and macrophage activation in lung tissue. **(A)** Levels of LPS in serum and lung tissue (n = 6). **(B)** Correlation heatmap between gut microbiota and lung glucose metabolism. **(C)** Correlation heatmap between gut microbiota and LPS (*P* < 0.05, Spearman’s correlation coefficient >0.3). **(D)** Correlation heatmap between LPS and lung glucose metabolism (*P* < 0.05, Spearman’s correlation coefficient >0.3). **(E)** Protein imprinting analysis of HIF-1a, CD80, and CD206 in lung tissue (n = 3). **(F)** Immunofluorescence staining of M1-type macrophages in lung tissue (200×). **(G-H)** Relative intensity ratio of the expressed M1-type macrophages (F4/80, CD80, n = 3). **(I)** Immunofluorescence staining of M2-type macrophages in lung tissue (×200). **(J-K)** The relative intensity ratio of the expressed M2-type macrophages (F4/80, CD206, n=3). **(L)** Detecting the proportions of M1-type and M2-type macrophages in lung tissue using flow cytometry (n = 8). *P < 0.05, **P < 0.01, ***P < 0.001, ****P < 0.0001.

Our findings revealed significant elevations in both serum and lung tissue LPS levels in the model group compared to the control group; however, after MXSG administration, these levels significantly decreased (Figure 5A). To explore the relationship between LPS, gut microbiota, and lung metabolic substances, we conducted Spearman’s correlation analysis. In Figures 5A, B correlation pattern between various microbial species and specific glycometabolites has been observed. Specifically, we found that *Rseburia* negatively correlates with glucose-6-phosphate and fructose-6-phosphate, *Ureibacilus* negatively correlates with dihydroxyacetone phosphate, while *Bifidobacterium, Butyricicoccus*, and *Facklamia* negatively correlate with 3-phosphoglyceraldehyde and phosphoenolpyruvate. Conversely, *Rikenella* and *Adlercreutzia* positively correlate with 3-phosphoglyceraldehyde and phosphoenolpyruvate. Figure 5C illustrates that LPS in serum and lung tissue exhibited a negative correlation with the genera *Rikenella*, *Lactobacillus*, and *Roseburia* and showed a positive correlation with the genera Prevotellaceae*_Prevotella*, *Akkermansia*, *Parabacteroides*, *Paraprevotella*, *Butyricicoccus*, *Buchnera*, *Sutterella*, *Oscillospira*, and *Allobaculum*. This suggests that an intricate interaction exists between gut microbiota and LPS in serum and lung tissue. Figure 5D shows that LPS in serum and lung tissue was positively correlated with lactate, pyruvate, glucose-6-phosphate, and fructose-6-phosphate. These results indicate a certain connection between LPS in serum and lung tissue, gut microbiota, and lung metabolic substances. MXSG possibly treats influenza virus-induced pneumonia by regulating gut microbiota, LPS, and lung tissue glucose metabolism.

Additionally, our findings suggest that HIF-1a can enhance glycolysis by activating glycolytic enzymes (Fulda and Debatin, 2007; Semenza, 2012; Xu et al., 2018), and glycolysis can promote M1-type macrophages, while oxidative phosphorylation can drive M2-type macrophages. Using Western blotting, we assessed the expression levels of HIF-1a, CD80, and CD206 proteins. Compared to the control group, the model group exhibited increased protein expression of HIF-1a and CD80, while CD206 protein expression decreased. After administering MXSG, HIF-1a, and CD80, protein expression decreased, and CD206 protein expression increased (Figure 5E). Immunofluorescence staining of lung tissues and flow cytometry analysis indicated an increase in M1-type macrophage activation in the lung tissue of influenza virus-induced pneumonia mice (Figures 5F–H, L) and a reduction in M2-type macrophage activation (Figures 5I–L). MXSG partially inhibited M1 macrophage activation (Figures 5F–H, L) and promoted M2 macrophage activation (Figures 5I–L). These results suggest that MXSG may regulate macrophage polarization by reducing HIF-1a expression, which is consistent with the earlier research results (Figure 3), which demonstrated the inhibition of glycolysis and promotion of oxidative phosphorylation.

## 4 Discussion

MXSG is a commonly used clinical treatment for respiratory infectious diseases (Yue and Ni, 1990; Chen et al., 2015). Research has demonstrated its multiple pharmacological mechanisms, including antiviral activity against influenza (Hsieh et al., 2012), inhibition of respiratory syncytial virus replication (Chen et al., 2015), and alleviation of acute lung injury (Zhu et al., 2023). Our previous studies have indicated that MXSG possesses antiviral properties, regulates immune-inflammatory responses, mitigates lung injury, and can modulate energy metabolism products in pneumonia-afflicted lung tissues (Li et al., 2020; Yang et al., 2020).

The relative content of MXSG of 18 major chemical metabolites exceeded 10,000,000. Of the 18 chemical metabolites, 9 were flavonoids. Earlier research suggested that flavonoids have the ability to hinder influenza and alleviate acute lung injury caused by H1N1(Bang et al., 2018; Ling et al., 2020; Yu et al., 2020). Ephedrine, the most abundant chemical metabolite in MXSG, can suppress the replication of influenza virus. Together, these studies suggest that the metabolites of MXSG have an anti-influenza effect. In this study, MXSG reduced the expression levels of pro-inflammatory cytokines TNF-α, IL-1β, and IL-6, elevated the expression level of the anti-inflammatory cytokine IL-10, improved lung tissue inflammatory injury, and demonstrated efficacy comparable to that of oseltamivir.

The gut microbiota play a critical role in maintaining normal immune function and ameliorating lung injury. The gut microbiota composition contributes to the regulation of the differentiation balance of CD4^+^ helper T lymphocytes, subsequently minimizing excessive inflammatory damage during the advanced stages of IAV infection (Ou et al., 2023). The oral administration of *Bacteroides* dorei elicited a rapid increase in the expression of type 1 IFNs during the initial phase of infection (on day 3), leading to a significant reduction in the viral load in the lungs. Conversely, during the later stages of infection (on day 7), it mitigated the levels of type 1 IFNs and other pro-inflammatory factors, thereby contributing to the enhancement of tissue repair processes (Song et al., 2021). Preserving the balance of gut microbiota is essential to maintaining the integrity of the intestinal mucosal barrier. This study demonstrated that influenza virus infection led to an increase in the abundance of *Rikenella*, Proteobacteria, and *Parabacteroides* and reduced the abundance of *Lactobacillus*. MXSG contributed to enhancing the relative abundance of *Clostridium, Candidatus Arthromitus* and *Lactobacillus* while reducing the relative abundance of Proteobacteria and *Parabacteroides* in the gut. *Clostridium orbiscindens*, as a specific species within the genus *Clostridium*, produces desaminotyrosine and protects against influenza by enhancing type I interferon signaling and reducing lung immunopathology (Steed et al., 2017). As one of the primary representatives of Gram-negative bacteria, the Proteobacteria phylum harbors bacterial outer membranes enriched with LPS components, contributing significantly as one of the major sources of gut-derived LPS. LPS also possesses the potential to further induce dysbiosis in the gut microbiota, subsequently leads to a significant increase in the abundance of the Proteobacteria phylum (Qiu et al., 2023). *Lactobacillus* can reinforce tight junction function and protect intestinal barrier function through various mechanisms such as inducing mucus production, regulating cell cytoskeleton, and phosphorylation of tight junction proteins (Sengupta et al., 2013). Therefore, we hypothesized that influenza virus infection may disrupt the gut microbiota structure, thereby damaging the intestinal mucosal barrier. MXSG may restore the physical barrier of the intestinal mucosa by increasing the abundance of beneficial bacteria such as lactic acid bacteria. Influenza virus infection increases the number of Proteobacteria and decreases the abundance of Firmicutes (Groves et al., 2018), which aligns with our research results. MXSG increased the abundance of Firmicutes and reduced the abundance of Proteobacteria, including *Parabacteroides* and *Helicobacter*.

The evidence demonstrates that specific microbial metabolites, such as N-acetyl-D-glucosamine (Hu et al., 2023), short chain fatty acids (Niu et al., 2023; Hu et al., 2024), and valine (Zhang et al., 2020), can alleviate influenza virus-induced acute lung injury through diverse mechanisms. Meanwhile, LPS derived from intestinal Gram-negative bacteria can induce the activation of inflammatory corpuscles in the lung, exacerbating inflammatory damage to lung tissues (Jin et al., 2021). Bacteroidetes is the largest class of bacteria among Gram-negative bacteria in the gut and is a major source of endogenous LPS (Ferro et al., 2020; Candelli et al., 2021). Our study results showed that influenza virus infection increased LPS levels in serum and lung tissue, while MXSG reduced LPS levels. Endogenous LPS levels are closely related to the degree of intestinal injury. When the intestinal mucosal barrier is damaged and permeability increases, LPS can transverse the intestinal mucosa, enter the bloodstream, and reach target organs through the circulatory system (Fu et al., 2022; Drummer et al., 2023). Therefore, we further examined the correlation between LPS and gut microbiota in this study. The results revealed that LPS in serum and lung tissue was positively correlated with *Parabacteroides* and negatively correlated with *Lactobacillus*. Therefore, we speculate that MXSG may restore the intestinal mucosal barrier function by increasing the abundance of *Lactobacillus* and reducing the abundance of Gram-negative bacteria such as *Parabacteroides*, thereby decreasing LPS in serum and lung tissue.

Different bacterial components and their metabolites can affect pulmonary glycolysis (Machado et al., 2022). We performed a correlation analysis between gut microbiota and glycolytic products, and the results showed that *Ureibacilus* negatively correlates with dihydroxyacetone phosphate. *Ureibacilus* belongs to the phylum *Firmicute*s. *Firmicutes* exhibits regulatory effects on the glycolysis (Zhang et al., 2024). LPS plays a significant role in promoting glycolysis in lung tissue. LPS mediates pulmonary tissue glycolysis through the regulation of the MKP-1/MAPK pathway (Li et al., 2023). Zhong et al. found that LPS mediated an increase in lung tissue glycolysis via HIF-1α, subsequently activating the NLRP3 inflammasome, thus promoting lung tissue injury (Zhong et al., 2023). We conducted a correlation analysis between LPS and glycolytic products, and the results showed that LPS was positively correlated with pulmonary glycolytic products such as lactate, pyruvate, glucose-6-phosphate, and fructose-6-phosphate, indicating a correlation between LPS and glycolysis. HIF-1a, a key protein regulating glycolysis (Wheaton and Chandel, 2011), maintains glycolytic flux by increasing the expression of glycolytic enzymes such as PKM2, pyruvate dehydrogenase kinase 1 (PDK1), and LDHA (Sun et al., 2019). In the context of influenza virus-induced pneumonia, there are notable interstitial changes characterized by exudation and thickening of alveolar septa. These changes restrict oxygen exchange, leading to an overexpression of HIF-1a induced by hypoxia. Under normoxic conditions, the influenza virus can elevate HIF-1a expression by impeding host cell proteasome function (Ren et al., 2019). In the present study, we observed an upregulation of HIF-1a expression in the lung tissue of mice with influenza virus-induced pneumonia, and MXSG administration reduced HIF-1a expression. Notably, influenza virus-induced glycolysis relies on the activation of HIF-1a (Ren et al., 2021). By inhibiting the glycolytic pathway, glucose metabolism can shift towards oxidative phosphorylation. Our study’s findings revealed an increase in the activity of glycolytic enzymes such as HK2, PKM2, and LDHA in lung tissue, along with elevated levels of glycolytic products, including glucose-6-phosphate, fructose-6-phosphate, and lactate. These results are consistent with prior research (Ritter et al., 2010; Petiot et al., 2011; Smallwood et al., 2017); however, the administration of MXSG inhibited glycolysis. LPS can enhance PKM2 activity and promote glycolysis by activating HIF-1a (Palsson-McDermott et al., 2015; Wang et al., 2019b). Therefore, it is plausible that the heightened intestinal LPS content following influenza virus infection, in conjunction with HIF-1a activation, alters host glucose metabolism, intensifying pulmonary glycolysis. MXSG, in turn, may regulate pulmonary glycolysis by reducing intestinal LPS content and inhibiting HIF-1a activity.

Targeting the gut microbiota can regulate the polarization of macrophages in the lung. The disruption of the gut microbiome and the proliferation of pathogenic bacteria can result in endotoxemia, leading to impaired functionality of intestinal mucosal epithelial cells, altered intestinal permeability, and subsequent translocation of a significant amount of endotoxins LPS passing through the blood to the lung. This process induces glycolytic reprogramming of macrophages, thereby regulating the polarization of macrophage (Palsson-McDermott, et al., 2015; Vallianou et al., 2021). Macrophages, primary inflammatory effector cells in the context of influenza virus infection, exhibit polarization into distinct subtypes in response to shifts in the external microenvironment. This polarization can occur rapidly and is highly reversible (Wang et al., 2020; Zhang et al., 2020). A fundamental event in the regulation of macrophage polarization is the metabolic reprogramming of glucose metabolism, which manifests as glycolysis-driven pro-inflammatory properties and oxidative phosphorylation-associated anti-inflammatory processes (O'Neill, 2015; Dayton et al., 2016). Our study results indicate that influenza virus infection propels the polarization of macrophages towards the M1 subtype, resulting in the upregulation of CD80 expression and heightened levels of pro-inflammatory cytokines such as TNF-a, IL-6, and IL-1β. Conversely, it suppresses the polarization of macrophages towards the M2 subtype, leading to the downregulation of CD206 expression and a reduction in the anti-inflammatory cytokine IL-10. Treatment with MXSG can modulate macrophage polarization, thereby ameliorating the inflammatory response in lung tissue during influenza virus infection. These findings suggest that MXSG may exert its effects by regulating macrophage polarization, ultimately mitigating the inflammatory response in lung tissue following influenza virus infection.

## 5 Conclusion

**FIGURE 6:**
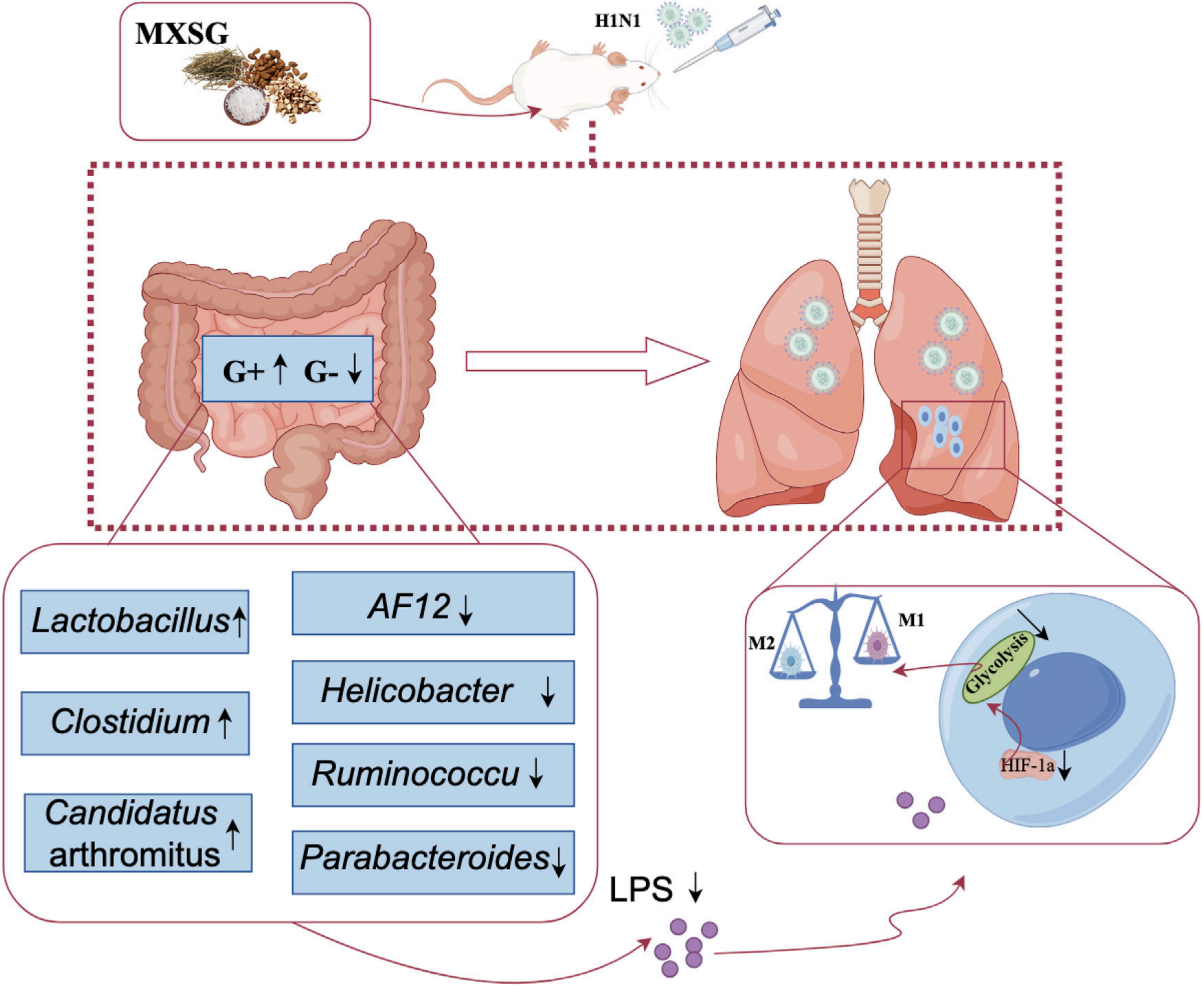
The mechanism of MXSG in treating influenza viral pneumonia.

Numerous respiratory infections coincide with an imbalance in the intestinal microbiota. Microbiome-sourced bacterial signatures and metabolites can be transmitted to the lung and could regulate pulmonary immune response (Liu et al., 2021). Treatments that target the gut microbiota can potentially prevent and treat respiratory diseases. MXSG can alleviate glycolysis in lung tissue, suppress M1-type macrophage activation, promote M2-type macrophage activation, and mitigate inflammation in lung tissue. This therapeutic effect appears to be mediated by modulating gut microbiota and reducing endogenous LPS production in the intestines (Figure 6); however, the optimal dosage and active metabolites of MXSG require further investigation. Additionally, incorporating clinical samples for further analysis will yield additional data to elucidate the mechanisms of MXSG in the treatment of influenza virus pneumonia for clinical applications.

## Data Availability

The data presented in the study are deposited in the NCBI repository, accession number PRJNA1138949.
