# Supplementary material for: Pharmacological mechanisms of Ma Xing Shi Gan Decoction in treating influenza virus-induced pneumonia: intestinal microbiota and pulmonary glycolysis
Source: Front Pharmacol. 2024 Aug 5;15:1404021. doi: 10.3389/fphar.2024.1404021 (PMC11331264; doi:10.3389/fphar.2024.1404021)
Supplement: Supplementary file 1 [file DataSheet1.pdf]

Three representative Total Ion Chromatograms of MXSG

1

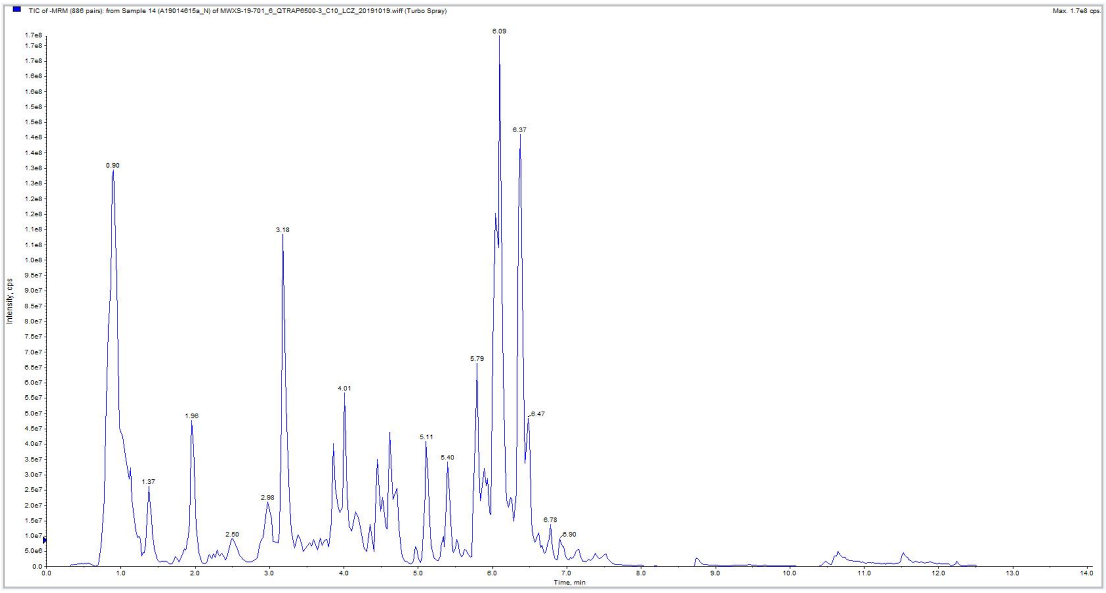

Positive ion chromatogram of MXSG

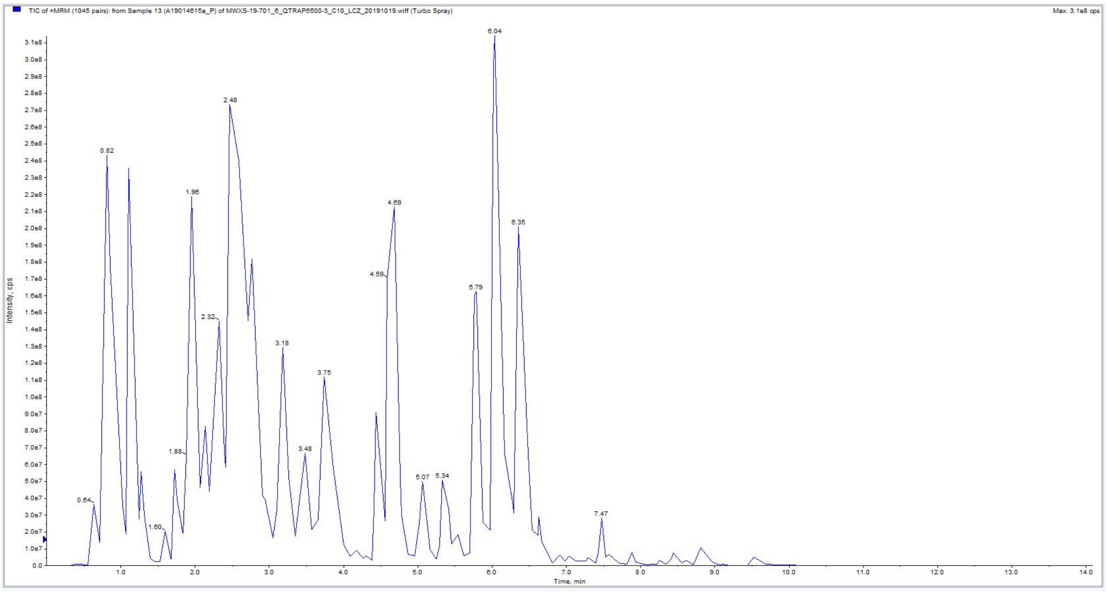

Anion chromatogram of MXSG UPLC-MS/MS.

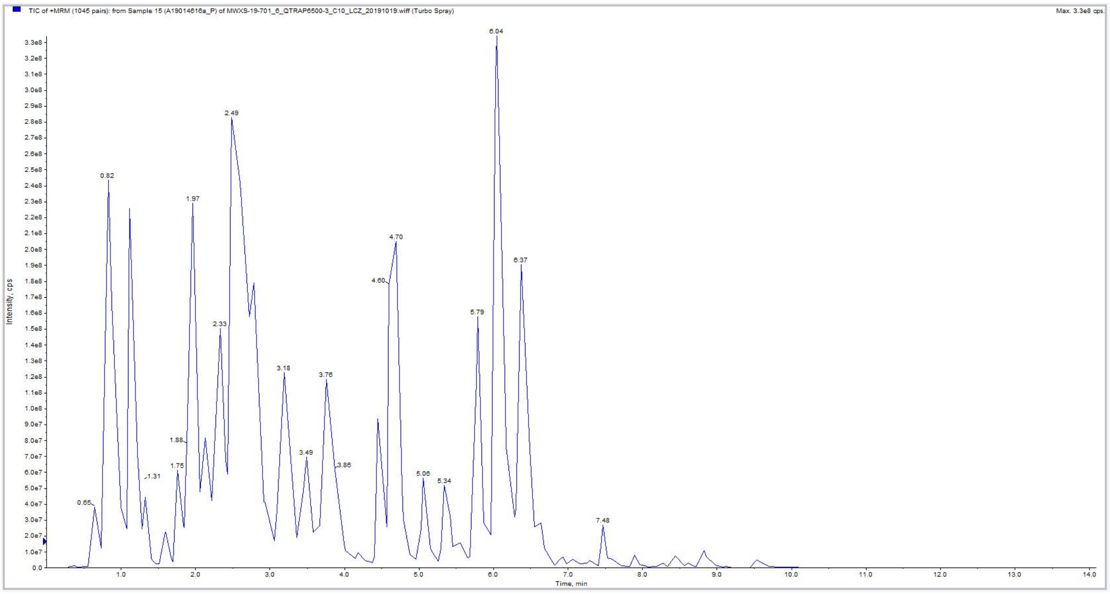

Positive ion chromatogram of MXSG

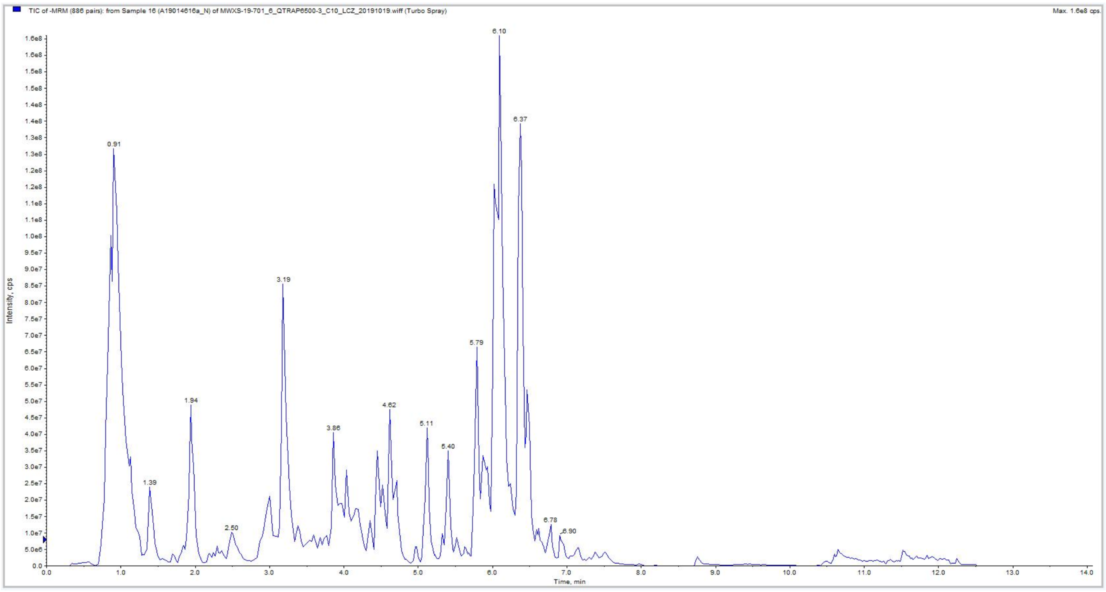

Anion chromatogram of MXSG UPLC-MS/MS.

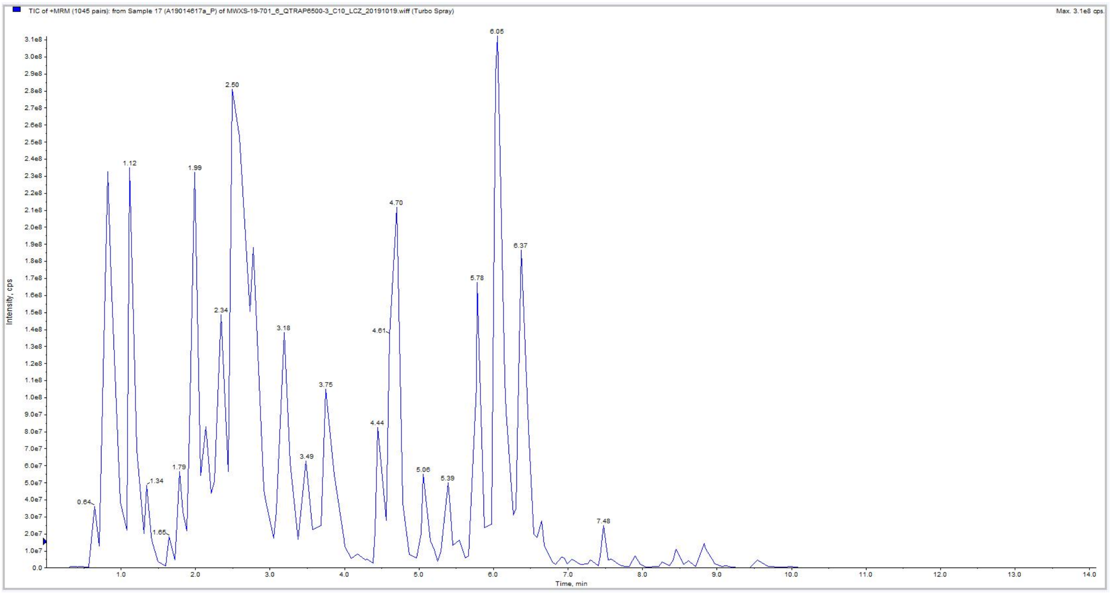

Positive ion chromatogram of MXSG

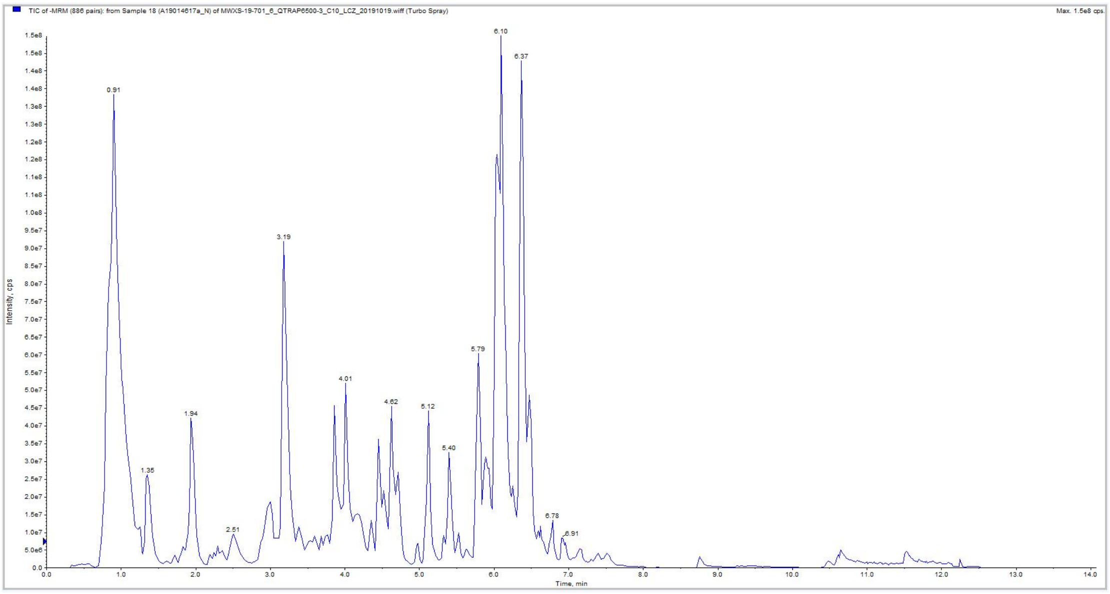

Anion chromatogram of MXSG UPLC-MS/MS.
